# Look What I Am Doing: Does Observational Learning Take Place in Evocative Task-Sharing Situations?

**DOI:** 10.1371/journal.pone.0043311

**Published:** 2012-08-14

**Authors:** Luca Ferraro, Cristina Iani, Michele Mariani, Roberto Nicoletti, Vittorio Gallese, Sandro Rubichi

**Affiliations:** 1 Department of Communication and Economics, University of Modena and Reggio, Emilia, Italy; 2 Department of Communication, University of Bologna, Bologna, Italy; 3 Department of Neuroscience, University of Parma, Parma, Italy; Katholieke Universiteit Leuven, Belgium

## Abstract

Two experiments were conducted to investigate whether physical and observational practice in task-sharing entail comparable implicit motor learning. To this end, the social-transfer-of-learning (SToL) effect was assessed when both participants performed the joint practice task (Experiment 1 – complete task-sharing), or when one participant observed the other performing half of the practice task (Experiment 2 – evocative task-sharing). Since the inversion of the spatial relations between responding agent and stimulus position has been shown to prevent SToL, in the present study we assessed it in both complete and evocative task-sharing conditions either when spatial relations were kept constant or changed from the practice to the transfer session. The same pattern of results was found for both complete and evocative task-sharing, thus suggesting that implicit motor learning in evocative task-sharing is equivalent to that obtained in complete task-sharing. We conclude that this motor learning originates from the simulation of the complementary (rather than the imitative) action.

## Introduction

It is well known that the ability to improve performance by observation, without physical practice, is a basic powerful human capacity (e.g. [1]–[6]). Even though researchers assessing the neuro-cognitive mechanisms supporting observational learning refer to this human ability by using different definitions (such as motor resonance, motor simulation, embodied simulation or mirroring; hereinafter we use the term motor simulation), they share the basic notion that action observation activates representations of corresponding motor programs in the observer's brain. The available empirical evidence suggests the existence of a basic neurophysiological system sub-serving this motor simulation. Single-cell recordings have indeed shown that some neurons, called mirror neurons, discharge both when a monkey performs or observes a given action [7], [8]. A similar mirror mechanism has been found in humans by means of functional magnetic resonance imaging (fMRI) investigations, by magnetoencephalography and by transcranial magnetic stimulation (TMS) studies (for review see [9], [10]).

All together, these findings suggest that when we observe another individual acting, our motor system in the brain simulates under threshold the perceived action and this simulation leads to motor facilitation for imitative behavior. However, many actions that we perform everyday require coordination with other people, rather than imitation of their actual action. If we think of simple situations, like dressing a child, or complex situations, like moving together with another person a heavy piece of furniture up a curvy staircase, it is clear that motor facilitation for imitative behavior would be most of the time detrimental for efficient performance. In these task-sharing situations (from now on, we will refer to them as “evocative task-sharing” situations), complementary rather than imitative acts are functional to properly reach a common goal.

There is some evidence that motor simulation in evocative task-sharing situations favors the activation of the complementary action, rather than of the imitative action. For instance, Newman-Norlund et al. [11] showed that the mirror mechanism is involved in planning both imitative and complementary actions depending on the task context. In their study, participants were required to observe a model grasping a manipulandum, either with a whole hand or with a precision grip, within an imitative or a complementary context. In the imitative condition, participants were asked to perform the observed action, while in the complementary condition participants had to execute the opposite grasp (for example, if they observed a precision grip they executed a whole hand grip). fMRI results indicated that action execution was facilitated by the observation of the identical action in the imitative context or by the observation of a different action in the complementary context. Such a finding is in line with the results by Ocampo and Kritikos [12] showing that during action observation the goal of the action and the context in which the action is performed shape the responses of our motor system. More recently, Sartori et al. [13], in a TMS study, showed that an observed action calling for an implicit complementary action might have the ability to prime non-identical responses, adding more evidence about a flexible context-dependent view of the action simulation system triggered by observation.

To summarize, there is some indication that observing an action in an evocative task-sharing situation triggers motor brain activations that may explain the motor facilitation effects found for the action that is complementary to the one that is observed. From these findings it may derive that observing an individual performing an action in an evocative task-sharing context would resemble acting in the very same context by performing the complementary action. In the present work, it was our intention to assess whether simulation of complementary actions in an evocative task-sharing condition, and actual performance of the same actions in a complete task sharing condition, translates into comparable motor learning effects. In other words, it was aimed at assessing whether physical and observational practice generate comparable motor learning effects.

A paradigm that seems particularly suited to test this prediction is the social version of the transfer of learning paradigm introduced by Milanese et al. [14], [15]. This paradigm, in which participants after practicing on a task are transferred to a similar task, was developed to study the effect produced by previous practice on the Simon task performed jointly by two participants ([16], see also [17], [18]). In the joint Simon task, two participants sitting in right-left positions respond to a non-spatial feature (for instance, color) of stimuli that are presented on the right or on the left of the screen (e.g., the left person responding to red stimuli, the right person responding to green stimuli). As when the task is performed by one single participant pressing two right-left keys ([19], for reviews see [20], [21]), performance is faster and more accurate when stimulus and response positions correspond (e.g., red stimulus requiring left response appearing on the left) than when they do not correspond (e.g., red stimulus requiring left response appearing on the right). The Simon task indexes the tendency to react to the same side of the source of stimulation, even if stimulus position is not relevant to select the correct response (e.g. [22]). It is considered a conflict task in which the response that spatially corresponds to stimulus position is automatically activated (e.g. [23]), likely because the stimulus is focused by spatial attention (e.g. [24]). In corresponding trials, this automatically activated response corresponds to the one indicated by task instructions and, as a consequence, performance is more efficient. Differently, in non-corresponding trials, the activated response interferes with the execution of the required response (e.g. [25], [26]). To note, in the individual Simon task the participant is required to perform binary choice responses (i.e., a choice RT task) while in the joint Simon task the participant is required to perform a single response (i.e., a go/no-go task) alongside another participant performing another single response (i.e., a complementary go/no-go task). Importantly, when the same go/no-go task is performed by a single individual, without the co-actor, no Simon effect normally emerges. It has been suggested that the emergence of the Simon effect when the task is distributed between two participants provides evidence that participants represented their co-actor's task and integrated their own and the other's actions in action planning (see [27] for a review, see also [28] for a critical review).

**Figure 1:**
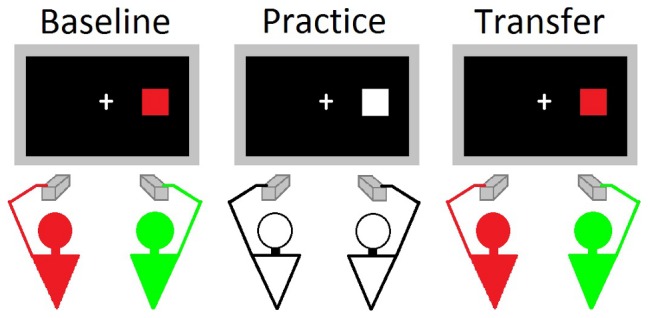
Schematic representation of the experimental conditions of the SToL paradigm used by Milanese et al. [**14**]. In the baseline session participants performed a joint Simon task, in the practice session they practiced with a joint spatially incompatible task, and in the transfer session they performed again the joint Simon task. A and B refer to the two participants.

Milanese et al. [14] found that, as for individual performance (e.g. [29], [30]), the joint Simon effect is modulated by prior practice. Specifically, if before performing the joint Simon task, participants perform a joint spatial compatibility task each responding to stimulus location by emitting a spatially incompatible response (that is, the participant on the left has to emit the left response to right stimuli, while the participant on the right has to emit the right response to left stimuli), the Simon effect is eliminated for both participants (that is, there is no difference between corresponding and non-corresponding trials). The disappearance of the Simon effect following a joint spatial compatibility task with an incompatible S-R mapping (see Figure 1), from now on referred to as social transfer of learning (SToL) effect, suggests that S-R associations acquired during joint physical practice are transferred to similar tasks, performed jointly.

Interestingly, Milanese et al. [15] found that there is not SToL effect if participants switch their sitting positions in between the practice and the transfer sessions, that is, when the participant sitting on the left in the practice session, carried out the joint Simon task sitting on the right and the participant sitting on the right in the practice session carried out the joint Simon task sitting on the left. This indicates that participants represent the joint spatial compatibility task performed during practice in terms of the spatial relations between themselves and the co-actor, as responding agents, and with respect to stimulus positions. If these specific S-R associations acquired during physical practice are not maintained during the subsequent Simon task, no SToL takes place.

In the present work, we conducted two experiments employing a modified version of the SToL paradigm (see Information S1). In both experiments, we compared a condition in which participants kept their sitting positions across tasks with a condition in which they switched position from one task to the other. In Experiment 1, that served to establish a reference baseline on the effect of physical practice, coupled participants performed together the practice and the transfer tasks. Based on previous studies [14], [15], we expected to find the SToL effect in the Non-Switch Condition only. In Experiment 2, we created an evocative task-sharing context by requiring to one of the participants in the couple to observe the other performing the practice task by responding to the contralateral stimulus in a go/no-go fashion. This context was evocative of a task-sharing context because, when the stimulus was presented in the contralateral position with respect to the observer, it called for her/his potential response. If observational practice, as physical practice, entails motor learning consequences, then, the observer should show a SToL effect. Furthermore, if observers during practice complete the task-sharing situation by simulating to perform the complementary action, then, in analogy to physical practice, the observer should not show a SToL effect if s/he switches sitting position with the agent after the practice session.

## Methods

### Participants

Forty-eight undergraduate students (36 female, age range 19–25 years) took part in Experiment 1, and thirty-six students (28 female, age range 19–24 years) took part in Experiment 2. Informed verbal consent was obtained from all participants after the nature and possible consequences of the study were explained to them.

All were naïve as to the purpose of the experiment and reported normal or corrected-to-normal vision. Once selected, they were randomly paired and each couple was randomly assigned to one of two experimental conditions (Non-Switch Condition or Switch Conditions). Since in Experiment 2 the evaluation of agents' performance in the Switch condition was unnecessary (because already assessed in Experiment 1), in this condition the agent was a confederate of the experimenter.

### Stimuli and Procedure

Stimuli consisted of solid squares (white in the spatial compatibility task, green and red in the Simon task) presented on a black screen, 9.5 cm to the left or to the right of a central fixation cross (1×1 cm). Participants sat in front of a PC monitor, at a viewing distance of about 70 cm. Stimuli presentation was controlled by E-Prime (Psychology Software Tools Inc. [31]). In both tasks, responses were executed by pressing the “z” or “–” key of a standard Italian keyboard with the left or right index finger, respectively.

Each experiment consisted of two consecutive sessions separated by a 5-min interval: a practice session and a transfer session. In the practice session participants were administered a spatial compatibility task with an incompatible S-R mapping, whereas in the transfer session they were administered a Simon task.

In both tasks, a trial began with presentation of the fixation cross at the center of a black background. After 1 sec the stimulus appeared to the right or to the left of fixation. In the spatial compatibility task, the stimulus remained visible for 600 ms and maximum time allowed for a response was 1200 ms. In the Simon task, the stimulus remained visible for 800 ms and maximum time allowed for a response was 1 sec. A response terminated the trial and the inter-trial interval was 1 sec.

**Figure 2:**
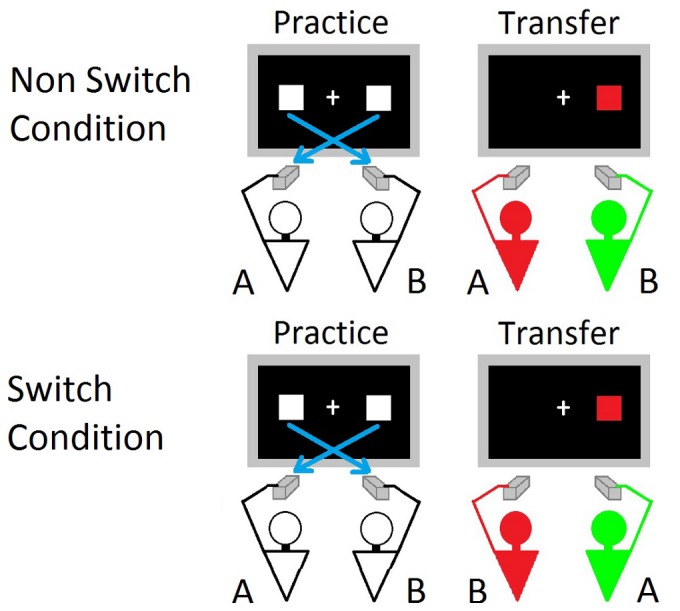
Schematic representation of the experimental conditions of the SToL paradigm used in the present work. In the practice session participants performed a joint spatially incompatible task, and in the transfer session they performed the joint Simon task. In the Non-Switch Condition participants kept the same sitting position from the practice to the transfer session, while in the Switch Condition they switched their sitting positions.

In Experiment 1 both tasks were performed jointly, in front of the same computer screen, with participants sitting alongside each other (see Figure 2). In the practice session participants were asked to respond to only one stimulus location by pressing the contralateral key: the participants sitting on the left responded to the right stimuli by pressing the left key with the left index finger, whereas the participants sitting on the right responded to the left stimuli by pressing the right key with the right index finger. In Experiment 2 only the Simon task was performed jointly. During the practice task only one participant for each pair was required to respond, while the other one was asked to watch carefully without emitting any response. More precisely, the agent was instructed to respond to contralateral stimuli (e.g., when the agent was seated on the right chair, s/he responded to left stimuli with the right key, whereas when the agent was seated on the left chair, s/he responded to right stimuli with the left key).

During the Simon task, each participant was instructed to respond to only one stimulus color. The mapping of the Simon task was balanced between participants.

For both experiments, in the Non-Switch Condition participants kept the same sitting positions across practice and transfer sessions, while in the Switch Condition participants exchanged their sitting positions after the practice session. In the Switch Condition of Experiment 2 the agent was a confederate of the experimenter.

The practice session consisted of 12 practice trials and 300 experimental trials that were divided into three blocks of 100 trials each; the transfer sessions consisted of 12 practice trials and 160 experimental trials that were divided into two blocks of 80 trials each.

## Results

For both experiments only the data of the Simon task were considered. Errors were very few and they were not further analyzed (Experiment 1: 1.1% for the Non-Switch Condition, 1.3% for the Switch Condition; Experiment 2: 0.8% for the Non-Switch Condition, 0.6% for the Switch Condition).

### Experiment 1

To assess the magnitude of the joint Simon effect in the two conditions, a repeated-measures analysis of variance (ANOVA) with Condition (Switch vs. Non-Switch) as a between-subjects factor and Correspondence (corresponding vs. non-corresponding) as a within-subject factor (see Table 1) was conducted on participants' reaction times (RTs). The Newman-Keuls test was used for all post-hoc comparisons.

**Table 1:** Agents' Performance in Experiment 1.

	Non-Switch Condition	Switch Condition
Corresponding	322 (63)	332 (62)
Non-corresponding	322 (64)	347 (66)
Simon effect	0	15*

Mean correct RTs in ms, standard deviations (in brackets) for corresponding and non-corresponding trials for the Non-Switch and Switch conditions of Experiment 1.The Simon effect was calculated by subtracting RTs on corresponding trials from RTs from non-corresponding trials (asterisk denotes significant differences).

The main effect of Correspondence was significant, F (1, 46) = 11.9, p<.01, η^2^_p_ = 20, as was the two-way interaction between Condition and Correspondence, F (1, 46) = 13.1, p <. 001, η^2^_p = _22. Post-hoc analyses showed that the difference between corresponding and non-corresponding trials (i.e., the Simon effect) was significant only for the Switch Condition (p<.001). A further analysis indicated that the performance of the two groups of agents in each condition did not differ (ps>.17).

### Experiment 2

A repeated measures ANOVA with Correspondence (corresponding vs. non-corresponding trials) as within-subject variable and Participant's role (agent in the Non-Switch condition, observer in the Non-Switch condition, and observer in the Switch condition) as between-subjects variable was conducted on RTs (see Table 2).

**Table 2:** Observers' Performance in Experiment 2.

	Non-Switch Condition	Switch Condition
Corresponding	345 (54)	320 (63)
Non-corresponding	350 (50)	338 (72)
Simon effect	5	18*

Mean correct RTs in ms, standard deviations (in brackets) for corresponding and non-corresponding trials for the Non-Switch and Switch conditions of Experiment 2. The Simon effect was calculated by subtracting RTs on corresponding trials from RTs from non-corresponding trials (asterisk denotes significant differences).

The main effect of Correspondence, F(1, 33) = 17.07, p<.001, η^2^_p_ = 34, was further modulated by Participant's role, as indicated by the significant two-way interaction, F(2, 33) = 4.74, p<.05, η^2^_p_ = 22. Post-hoc analyses showed that the difference between corresponding and non-corresponding trials (i.e., the Simon effect) was significant only for the observers who switched position across practice and transfer session (p<.001). Neither the agent (340 vs. 344 ms for corresponding and non-corresponding trials, respectively) nor the observer (345 vs. 350 ms for corresponding and non-corresponding trials, respectively) in the Non-Switch condition showed a significant Simon effect. A follow-up analysis showed that the 4-ms Simon effect shown by the agents and the 5-ms Simon effect shown by the observers did not differ (p = .73). Hence, learning effects deriving from observing a practice task in an evocative task-sharing context do not seem to differ from those deriving from actually performing a joint practice task.

### Comparison between the two experiments

A possible way to assess whether the data pattern in the two experiments is equivalent would be to perform an equivalence test [32]. Equivalence tests require to make an a priori decision concerning the minimum difference between two groups that would be important to make the groups nonequivalent. However, since in our case this minimum difference would be the result of a completely arbitrary decision, we decided to use a differ analysis. Specifically, we assessed whether the effect of the interaction between condition (Non-Switch vs. Switch) and correspondence (indicative of the elimination of the Simon effect in the non-switch condition only) differed between the two experiments. To this end, all participants' RTs were submitted to a repeated measures ANOVA with Experiment and Condition (non-switch vs. switch) as between-subjects factors and Correspondence as within-subject factor.

This analysis showed a main effect of Correspondence, F(1, 80) = 32.26, p<.001, η^2^_p_ = 29, and a significant interaction between Correspondence and Condition, F(1,80) = 21.10, p<.001, η^2^_p_ = 21. Crucially, this two-way interaction was not further modulated by Experiment, F<1, hence indicating that the data pattern emerging form the interaction between Condition and Correspondence did not differ between the two experiments. This result clearly demonstrates that the SToL effect found in the Non-Switch Condition does not differ between Experiments.

## Discussion

This study was aimed at assessing observational learning occurring in an evocative task-sharing context in which an individual observes another individual performing her/his half of the task. We reasoned that observing someone else performing an action that calls for a complementary action should bring to the same effects evident when actually performing the action complementary to the observed action. If this is the case, observational practice in evocative task-sharing should generate motor learning effects comparable to those obtained after physical practice.

Two experiments employing the SToL paradigm [14], [15] were designed to test this prediction. In Experiment 1 both participants performed jointly the practice session, while in Experiment 2 one participant observed a confederate performing half of the practice session. This was the only difference between the two experiments. In both experiments, for the reason exposed above, the control condition was when participants switched their sitting positions in between the practice and the transfer sessions. In this case, no SToL was expected.

Experiment 1 clearly replicated the pattern of results obtained in previous studies: SToL was present only when participants kept their sitting position across sessions (Non-Switch Condition). When they switched sitting positions (Switch Condition) a regular 15-ms joint Simon effect was obtained. In line with Milanese et al. [15], we interpreted this result as an indication that participants represented the joint spatial compatibility task in terms of the spatial relations between themselves and the co-actor. If these spatial relations were kept constant in the subsequent Simon task, then there was SToL. On the contrary, when participants' position changed from the practice to the transfer task (Switch Condition), the SToL effect did not occur, suggesting that participants' representations of the practice and transfer tasks did not overlap enough to permit SToL. These results also suggest that during the practice session participants did not implicitly acquire an abstract response selection strategy based on an “emit the alternative response” rule, as it happens when the transfer of learning is studied in individual performance [33]. If this were the case, they should have shown a modulation of the Simon effect irrespective of the change of spatial relations caused by the change of sitting positions. Thus, provided that task spatial relations are invariant across tasks, what is possibly acquired and transferred is the link between specific S-R spatial features.

According to our predictions, in Experiment 2 we expected to find a) a SToL effect in the observer but b) the effect should not arise if s/he switches sitting position with the agent after the practice session. Both these predictions were confirmed: the observer showed SToL when s/he kept the same sitting position across the practice and the transfer tasks (i.e., Non-Switch Condition), while no evidence of SToL was found when the observer switched sitting position from the practice to the transfer task (i.e., Switch Condition).

On the whole, these findings suggest that in an evocative task-sharing context, the observer implicitly learns S-R associations and transfers this motor learning to similar tasks. Indeed, we found clear evidence that the motor simulation mechanism activates motor representations to complete task-sharing by performing the complementary action. Interestingly, the agent also showed SToL even when the other participant simply observed the practice task, while previous investigations found no evidence of transfer of learning when an agent performed the go/no-go practice task alone [14]. This means that also the agent's representation of the task includes the observer as a potential partner responding to one stimulus. Thus, in evocative task-sharing situations, the representation of the other's task, which leads to the integration of one's own and of the other's actions in action planning, does not necessitate that both individuals actually perform their half of the task.

To summarize, the present results provide further evidence of how flexible and context-dependent the motor simulation evoked by observed actions can be. Sartori et al. [13] demonstrated that observed actions calling for an implicit complementary response might prime complementary actions. Our findings extend their results to task-sharing situations, demonstrating that the observation of an action calling for a complementary action, similarly to actual performance, translates into motor learning. According to Sartori et al. [13], observation of an action that calls for a complementary action leads initially to an automatic simulation of the observed action in order to experience and understand what is observed, then the complementary action is activated. The results of the present study support this view, by showing evidence of motor learning deriving from the simulation of the complementary action.

The present results have important practical implications. The understanding of the mechanisms underlying observational learning and of the conditions in which it occurs is particularly valuable because observational learning is fundamental not only for acquiring skills in everyday life but also for neuromotoric rehabilitation in a variety of medical conditions causing the loss or limitation of motor abilities. In recent years, the discovery that action observation activates the same cortical motor areas that are involved in the execution of the observed actions has led to the development of a new rehabilitative approach, called Action Observation Therapy, that consists in asking patients to observe, for instance, video clips showing daily actions and to imitate them afterward. For instance Ertelt et al. [34] found that the observation of everyday purposeful actions matched with physical practice led to a significant improvement of motor functions that lasted for at least 8 weeks after the end of the intervention. This improvement was significantly higher than deriving from physical training alone. In general, Action Observation Therapy is based on the imitation of observed individual actions. Our results suggest that it might be also used in joint contexts to train the appropriate complementary actions. Understanding when and what humans learn while observing the actions of others may help in identifying which motor disabilities may gain benefit from the application of therapies based on action observation. Future research should be directed at generalizing the conclusions derived from the present study by employing tasks involving more complex motor abilities and by evaluating the role of motor competencies and of the intention to learn.

## Supporting Information

Supporting Information S1 **Description of the SToL paradigm used by Milanese et al.** [**14**]. The SToL paradigm used in the present study slightly differed from the version originally developed by Milanese et al. [14]. In the original paradigm, coupled participants performed jointly three consecutive sessions. In the first session (baseline) participants performed a joint Simon task, in the second session (practice session) they performed a spatial compatibility task with an incompatible mapping between stimulus and response, while in the third session (transfer session) they performed again the joint Simon task (see Figure 1). Since the present work was focused on observational learning in task-sharing, we thought it important to test the SToL of observers who had not performed the Simon task at baseline, that is, had no prior experience of task-sharing during the execution of the Simon task. For this reason, no baseline session was included.
(DOCX)
