# Supplementary material for: Look What I Am Doing: Does Observational Learning Take Place in Evocative Task-Sharing Situations?
Source: PLoS One. 2012 Aug 14;7(8):e43311. doi: 10.1371/journal.pone.0043311 (PMC3419169; doi:10.1371/journal.pone.0043311)
Supplement: Supporting Information S1 — Description of the SToL paradigm used by Milanese et al. [14]. The SToL paradigm used in the present study slightly differed from the version originally developed by Milanese et al. [14]. In the original paradigm, coupled participants performed jointly three consecutive sessions. In the first session (baseline) participants performed a joint Simon task, in the second session (practice session) they performed a spatial compatibility task with an incompatible mapping between stimulus and response, while in the third session (transfer session) they performed again the joint Simon task (see Figure 1). Since the present work was focused on observational learning in task-sharing, we thought it important to test the SToL of observers who had not performed the Simon task at baseline, that is, had no prior experience of task-sharing during the execution of the Simon task. For this reason, no baseline session was included. (DOCX) [file pone.0043311.s001.docx]

**Supporting Information S1 – Description of the SToL paradigm used by Milanese et al. [14]**

The SToL paradigm used in the present study slightly differed from the version originally developed by Milanese et al. [14]. In the original paradigm, coupled participants performed jointly three consecutive sessions. In the first session (baseline) participants performed a joint Simon task, in the second session (practice session) they performed a spatial compatibility task with an incompatible mapping between stimulus and response, while in the third session (transfer session) they performed again the joint Simon task (see Figure 1). Since the present work was focused on observational learning in task-sharing, we thought it important to test the SToL of observers who had not performed the Simon task at baseline, that is, had no prior experience of task-sharing during the execution of the Simon task. For this reason, no baseline session was included.
